# Structural Coupling of SH2-Kinase Domains Links Fes and Abl Substrate Recognition and Kinase Activation

**DOI:** 10.1016/j.cell.2008.07.047

**Published:** 2008-09-05

**Authors:** Panagis Filippakopoulos, Michael Kofler, Oliver Hantschel, Gerald D. Gish, Florian Grebien, Eidarus Salah, Philipp Neudecker, Lewis E. Kay, Benjamin E. Turk, Giulio Superti-Furga, Tony Pawson, Stefan Knapp

**Affiliations:** 1 Structural Genomics Consortium, University of Oxford, Old Road Campus, Roosevelt Drive, Oxford OX3 7DQ, UK; 2 Samuel Lunenfeld Research Institute, Mount Sinai Hospital, 600 University Avenue, Toronto, Ontario M5G 1X5, Canada; 3 Center for Molecular Medicine of the Austrian Academy of Sciences, Lazarettgasse 19, 1090 Vienna, Austria; 4 Department of Biochemistry, University of Toronto, Toronto, Ontario M5S 1A8, Canada; 5 Yale University School of Medicine, Department of Pharmacology, New Haven, CT 06520, USA; 6 Department of Molecular Genetics, University of Toronto, Toronto, Ontario M5S 1A8, Canada; 7 Department of Clinical Pharmacology, University of Oxford, Old Road Campus, Roosevelt Drive, Oxford OX3 7DQ, UK

**Keywords:** CELLBIO, SIGNALING, PROTEINS

## Abstract

The SH2 domain of cytoplasmic tyrosine kinases can enhance catalytic activity and substrate recognition, but the molecular mechanisms by which this is achieved are poorly understood. We have solved the structure of the prototypic SH2-kinase unit of the human Fes tyrosine kinase, which appears specialized for positive signaling. In its active conformation, the SH2 domain tightly interacts with the kinase N-terminal lobe and positions the kinase αC helix in an active configuration through essential packing and electrostatic interactions. This interaction is stabilized by ligand binding to the SH2 domain. Our data indicate that Fes kinase activation is closely coupled to substrate recognition through cooperative SH2-kinase-substrate interactions. Similarly, we find that the SH2 domain of the active Abl kinase stimulates catalytic activity and substrate phosphorylation through a distinct SH2-kinase interface. Thus, the SH2 and catalytic domains of active Fes and Abl pro-oncogenic kinases form integrated structures essential for effective tyrosine kinase signaling.

## Introduction

Protein domains are modules that can be joined in new combinations during evolution to create novel cellular functions. Once covalently tethered, such domains can acquire specific intramolecular interactions that yield selective allosteric regulation (Kuriyan and Eisenberg, 2007). These processes have been at work in the evolution of multidomain cytoplasmic tyrosine kinases. Most of these enzymes have a core unit comprised of a protein kinase domain and an adjacent Src homology 2 (SH2) domain (Figure S1 available online), an ancient combination that likely emerged at the dawn of phosphotyrosine (pTyr) signaling in organisms such as *Dictyostelium discoideum* (Eichinger et al., 2005). A likely role of this ancestral fusion of SH2 and kinase domains was to enhance the phosphorylation of specific substrates (Moniakis et al., 2001). In metazoans, the SH2-kinase core is typically flanked by additional regulatory domains, such as SH3. In addition to their positive role in kinase activity and substrate recognition, the interaction domains of tyrosine kinases such as Src, Abl, and ZAP-70 have also acquired an ability to suppress catalytic activity through intramolecular interactions. The structural basis for these autoinhibitory effects has been analyzed in detail (Deindl et al., 2007; Nagar et al., 2003; Sicheri et al., 1997; Xu et al., 1997). However, we lack a corresponding understanding of the mechanisms by which the SH2 domain synergizes with the tyrosine kinase domain in the active state.

The Fps/Fes cytoplasmic tyrosine kinase was identified as a transforming protein encoded by avian (*Fps*) and mammalian (*Fes*) retroviral oncogenes, in which retroviral Gag sequences are fused to the N termini of cell-derived Fps/Fes products (Groffen et al., 1983; Lee et al., 1980; Shibuya et al., 1980). We previously used insertion mutagenesis to identify domains required for Fps catalytic and biological activities in the context of the P130^gag-fps^ viral (v−) oncoprotein of Fujinami avian sarcoma virus (Sadowski et al., 1986; Stone et al., 1984). This approach revealed three regions that are important for v-Fps transforming activity: the C-terminal tyrosine kinase domain, an adjacent sequence designated as the SH2 domain that modifies kinase activity and substrate recognition, and an N-terminal domain (Nfps) involved in membrane localization (Brooks-Wilson et al., 1989), now annotated as a member of the extended FCH or F-BAR domain family (Itoh and De Camilli, 2006).

Fps/Fes proteins do not possess an SH3 domain and, consequently, are not autoinhibited through intramolecular interactions in the fashion of Src and Abl tyrosine kinases (Greer, 2002). In contrast, they may be regulated by reversible membrane association and oligomerization mediated by the F-BAR domain and by recruitment to specific subcellular sites by the SH2 domain (Brooks-Wilson et al., 1989; Naba et al., 2008; Read et al., 1997; Tsujita et al., 2006). The N-terminal Gag sequences of v-Fps/Fes oncoproteins result in more stable membrane localization and, consequently, in constitutive autophosphorylation (Foster et al., 1985; Greer et al., 1994). For both v-Fps (Weinmaster et al., 1984) and Fes (Hjermstad et al., 1993), autophosphorylation in the kinase domain stimulates catalytic activity.

Dipeptide insertions in the N-terminal region of the v-Fps SH2 domain cause a severe loss of both kinase and transforming functions (DeClue et al., 1987; Sadowski et al., 1986). Taken with the observation that the SH2 and kinase domains form a 45 kDa protease-resistant fragment (Koch et al., 1989; Weinmaster and Pawson, 1982), such data have suggested that the v-Fps SH2 domain undergoes an intramolecular interaction with the kinase domain that stimulates catalytic activity. The SH2 domains of v-Fps and human Fes also aid in substrate recognition (DeClue et al., 1987; Jucker et al., 1997; Koch et al., 1989), potentially by binding pTyr sites on primed targets. Fps/Fes proteins, therefore, provide a model to study the cooperative effects of interaction and catalytic domains in promoting the kinase active state.

Mammals have two proteins with an F-BAR-SH2-kinase domain architecture, Fes and Fer. These have a range of functions, including regulation of adherens junction stability, actin dynamics, vesicular trafficking, and receptor internalization, mediated by phosphorylation of substrates such as cortactin, Pecam-1, and β-catenin (Greer, 2002; Sangrar et al., 2007; Udell et al., 2006). In vivo, murine Fes and Fer regulate hemostasis and innate immunity (Greer, 2002; Parsons and Greer, 2006; Parsons et al., 2007).

Here, we identify the molecular mechanisms by which the human Fes SH2 and kinase domains interact with one another and with substrate to promote the active state. We show that the linked SH2 and catalytic domains of the active Abl tyrosine kinase, like Fes, act as a unit in which the SH2 domain stimulates the adjacent kinase domain. These results show how tyrosine kinase activity can be coupled to substrate recognition and identify a common strategy by which the SH2 domains of distinct cytoplasmic tyrosine kinases promote substrate phosphorylation.

## Results

### Determination of the Fes SH2-Kinase Structure

**Figure 1:**
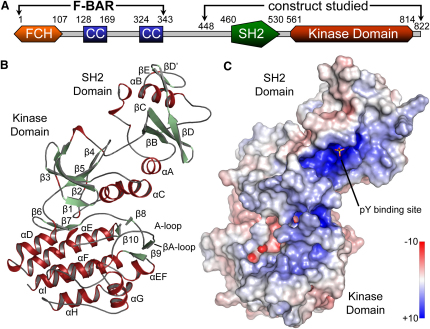
Structural Overview of Human Fes (A) Domain architecture. The locations of the F-BAR, SH2, and kinase domains are indicated. The F-BAR domain contains the FCH and coiled-coil (CC) sequences. The crystallized construct is highlighted by arrows. (B) Ribbon diagram representing the overall structure of the SH2-kinase domain unit. The main secondary structure elements are labeled. (C) Surface representation of active Fes. The surface is colored by electrostatic potential between −10 and +10 kcal/mol.

We investigated the structure of a polypeptide from human Fes comprising a linker region N terminal to the SH2 domain, the SH2 domain, and the C-terminal kinase domain (Figure 1A). We obtained high yields for a single Fes construct (containing residues I448 to the C terminus) in *E. coli* coexpressed with the YopH tyrosine phosphatase (Seeliger et al., 2005). To explore the mechanisms of kinase regulation and substrate recognition, we solved structures of the unphosphorylated Fes SH2-kinase unit both in the presence and absence of a kinase consensus substrate peptide. In addition, we analyzed Fes SH2-kinase that had been autophosphorylated on the kinase activation segment (Y713) in complex with a substrate peptide. The three structures were determined in complex with the ATP mimetic kinase inhibitor staurosporine and were refined to low R factors and acceptable geometry (Table S1). The binding mode of staurosporine to the ATP pocket is similar to structures reported previously (Bertrand et al., 2003); in addition, two molecules of staurosporine were identified in crystal contacts in Fes-substrate complexes. The initial structure of unphosphorylated Fes SH2-kinase revealed an active conformation, which was stabilized by two sulphate ions that mimic binding of a pTyr ligand to the SH2 domain, as well as activation segment phosphorylation. This structure was well ordered, showing productive interactions between the SH2 and kinase domains, and we were able to trace the entire chain; we refer to this structure as “active.” In contrast, the other two structures have no ligand bound to the SH2 domain and show disordered loop regions in the SH2 domain and in the upper kinase lobe, indicative of an inactive conformation. Composite analysis of all three structures indicates multiple mechanisms for ordering of the activation segment.

### Structural Overview of an Active Fes Conformation

**Figure 2:**
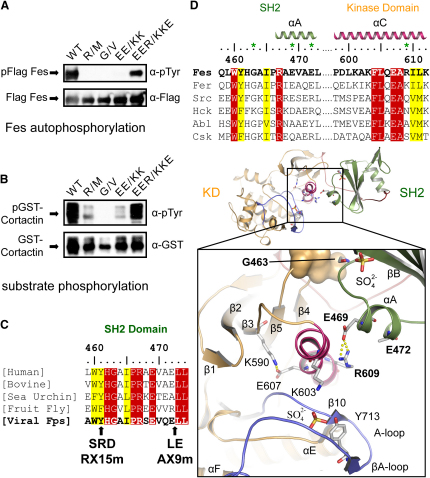
Activation of Fes Kinase Activity by SH2 Domain Interactions (A) The effect of mutations in the SH2 domain on Fes kinase activity: HEK293T cells were cotransfected with Flag-tagged Fes and GST-cortactin. Cell lysates were immunoprecipitated with anti-Flag and blotted with anti-pTyr to reveal autophosphorylated (p) Flag-Fes (top panel) or with anti-Flag (lower panel) as a control. WT indicates wild-type Fes, and mutants are described in the text. (B) GST-cortactin was affinity purified from cells coexpressing WT or mutant Fes proteins and blotted either with anti-pTyr antibodies to measure Fes-induced phosphorylation (p) (top panel) or with anti-GST (bottom panel). (C) Comparison of the N-terminal SH2 domain sequence of Fps/Fes orthologs. Insertions originally used to identify the SH2 domain in v-Fps are indicated (RX15m and AX9m) (Sadowski et al., 1986; Stone et al., 1984). Conserved residues are highlighted in red and similar residues in yellow. (D) Details of the Fes SH2-kinase interface. Residues important for the SH2 domain-kinase interaction are conserved in Fps/Fes family members but not in other tyrosine kinases. Residues mutated in this study are indicated by a green asterisk. Interactions of interface residues and the involved secondary structure elements are shown in the structure of active Fes. The alignment of human sequences is colored as in (C).

**Figure 3:**
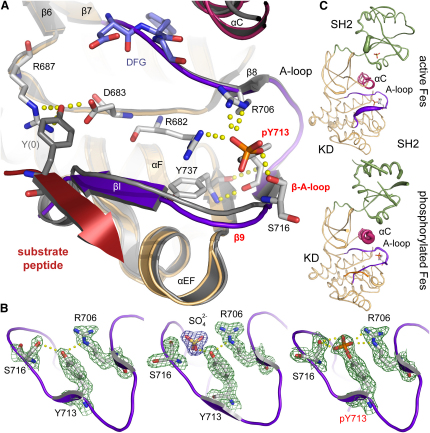
Conformation of the Fes Activation Segment (A) Superimposition of the activation segment in active Fes (gray) and the phosphorylated Fes-substrate complex. Hydrophilic interactions formed by the phosphate moiety and the substrate tyrosine are indicated by yellow dots. The antiparallel β sheet formed at the tip of the activation segment is labeled as β-A-loop, and the substrate peptide is shown as a red ribbon. The induced β sheet present in the substrate complex (βI) is also shown. (B) Interactions of the activation segment Y713 in unphosphorylated (left), active (ligating a sulphate ion; middle), and phosphorylated (right) Fes. A 2F_o_ – F_c_ electron density map around the interacting residues is also shown contoured at 2σ. (C) Schematic drawing of the Fes main chain. Regions with high temperature factors are shown by an increasing radius of the backbone. The activation segment is highlighted in purple; αC, in pink; the SH2 domain, in green; and the substrate peptide (in the phosphorylated Fes structure), in orange. Note the absence of ordered loop regions in the SH2 domain and kinase N lobe of phosphorylated Fes.

An overview of unphosphorylated Fes SH2-kinase in its catalytically active conformation is shown in Figure 1B. In this active configuration, the SH2 domain is stabilized by a sulphate ion, which mimics the phosphate of a pTyr ligand by coordinating a conserved SH2 domain arginine (R483), as well as R467 and S485 (see Figure 5A). In addition, a sulphate ion is coordinated by Y713 and R706 in the activation segment, thereby mimicking autophosphorylation (see Figure 3B). The SH2 and kinase domains form a stable unit linked by the N-terminal region of the SH2 domain and polar interactions between the SH2 domain helix αA and the catalytically important helix αC (Figures 1C, 2D, and S2). In Fes/Fer family members, the linker region between the SH2 and kinase domains is about 8 residues shorter when compared to other cytoplasmic tyrosine kinases, constraining the packing of the SH2 domain. The N terminus of the SH2 domain (^462^HGAI) intercalates between the central SH2 β sheet and the loop region between strands β4 and β5 in the kinase domain (Figures 2D and S3). This tight packing does not leave room for bulky side chains, and the central glycine residue is present in all Fes family members, suggesting that this domain packing is conserved.

The second major interaction site is formed by a network of acidic residues located in the SH2 helix αA (E469, E472) and R609 in αC of the kinase. This arrangement suggests that the interaction between the SH2 and kinase domains positions and stabilizes αC in an active conformation, as indicated by the salt bridge formed between the active site lysine (K590) and the conserved αC glutamate (E607), as well as by low B values in loop regions linking this helix (Figure 2D). The structure, therefore, shows the SH2 domain forming an extensive interface with the N lobe of the kinase domain that promotes the organization of a functional kinase active site.

### A Tight SH2-Kinase Interaction Is Required for Fes Activity

To pursue the relevance of the observed interdomain interactions, we studied the effects of mutating residues in the SH2-kinase interface on human Fes activity. To this end, we monitored Fes autophosphorylation and transphosphorylation activity in transfected cells, using cortactin (residues 382–550) fused to GST as an exogenous substrate (Figures 2A and 2B). In an effort to perturb the close interaction between the SH2 and kinase domains, we mutated the conserved glycine residue (G463) located in the tightly packed interface between the SH2 N terminus and the kinase β4/β5 loop to valine (mutant G/V). Introducing this bulky side chain would be expected to sterically hinder alignment of the SH2 and kinase domains, resulting in an altered domain packing. Wild-type (WT) and G/V mutant full-length human Fes, each with an N-terminal Flag epitope, were expressed in HEK293T cells together with GST-cortactin. The WT protein was active as measured by autophosphorylation and tyrosine phosphorylation of cotransfected cortactin, whereas the G/V Fes mutant was completely inactive (Figures 2A and 2B).

To extend this approach, we mutated residues involved in the salt bridge network, which appear to position and stabilize the kinase N-terminal lobe helix αC. In particular, we mutated E469 and E472 in the SH2 domain αA helix to lysine either individually or together (mutant EE/KK). The Fes E469K mutant exhibited reduced tyrosine kinase activity, whereas the E472K mutant was not significantly impaired (data not shown). Consistent with this finding, the side chain of E472 was not visible in the electron density and was assumed to be unstructured. However, mutation of both E469 and E472 to lysine strongly reduced Fes kinase activity, suggesting that both side chains contribute synergistically to this electrostatic interaction (Figures 2A and 2B). This is consistent with the observation that the SH2-kinase domain interface shows a charge complementarity such that the surface of the SH2 domain interacting with the kinase is mainly negatively charged, whereas the corresponding kinase domain surface is positively charged (Figure S2). To demonstrate that these mutations did not disturb the structural integrity of Fes, we sought to rescue the inactive double mutant EE/KK by inverting the polarity of the interface by simultaneously mutating R609 in the αC helix to glutamate. Indeed, the triple mutant (mutant EER/KKE) regained kinase activity to a level approaching wild-type (Figures 2A and 2B), providing direct evidence that the electrostatic interactions at the SH2-kinase interface are functionally important. In support of the notion that the G/V and EE/KK mutations have a selective effect on the SH2-kinase interface, they did not alter the affinity of the isolated Fes SH2 domain for a pTyr-containing peptide from ezrin (VpYEPVSY). The SH2-kinase polypeptide had a similar affinity for the ezrin phosphopeptide as did the isolated SH2 domain (Figure S4).

We introduced the same mutations into a Myc-tagged P130^gag-fps^ oncoprotein and found that a G823V substitution, analogous to the G/V mutation in Fes, completely inhibited kinase activity (Figure S5). Of interest, the inactivating RX15m v-Fps insertion that was isolated in the earlier mutagenesis screen changes the tyrosine located 2 residues N terminal to G823 to SRD and, therefore, likely inhibits kinase activity by destabilizing the packing between the N terminus of the SH2 domain and the β4/β5 loop of the kinase domain (Figures 2C and S5). Single or double mutations of E829 and E832 to lysine or alanine showed similar, albeit less pronounced, effects as the corresponding substitutions in Fes (at E469 and E472), and the activity of an EE/KK mutant was restored by further substitution of E for R969 (EER/KKE), equivalent to R609 in the Fes kinase αC helix (data not shown). Supporting the importance of this interface in v-Fps, the AX9m mutation in v-Fps inserts a LE dipeptide between E832 and L833 in the SH2 αA helix, which likely disturbs the electrostatic interface between the SH2 domain and kinase αC helix. Thus, the RX15m and AX9m v-Fps insertion mutations that originally identified the SH2 sequence as a functional module map to the two primary elements that interact with the kinase domain. Therefore, the SH2-kinase interface revealed by structural analysis of human Fes is also critical for the transforming activity of the v-Fps oncoprotein. In summary, the residues at the SH2-kinase domain interface stabilize an active conformation of the Fes kinase domain, in part by positioning and stabilizing helix αC to form the active site.

### Substrate Binding and an Antiparallel β Sheet Stabilize the Fes Activation Segment

To better understand Fes regulation, we also solved structures of unphosphorylated Fes in the absence of phosphomimetic salt ions and Fes in which the regulatory activation segment residue (Y713) had been autophosphorylated in vitro (Table S1). In both cases, a substrate peptide (IYESL) was cocrystallized. As expected, in phosphorylated Fes, the phosphorylated Y713 formed a network of salt bridges and hydrogen bonds of the sort typically observed in activation segments of activated kinases (Figure 3A). This links the conserved catalytic loop arginine (R682) with the activation segment and additionally stabilizes the active conformation through hydrogen bonds to R706 and S716. These polar interactions are mimicked by the sulphate ion present in active Fes (Figure 3B). Interestingly, the activation segment of unphosphorylated Fes crystallized in the absence of sulphate ions assumed a similar conformation, stabilized by hydrogen bonds to the Y713 hydroxyl group, suggesting that the Fes activation segment is quite stable in the absence of tyrosine phosphorylation.

Two structural features contribute to this stability. First, the activation loop forms a short antiparallel β sheet with the loop region linking the helices αEF with αF in the kinase C lobe. Interestingly, this β sheet is present in most active (phosphorylated) tyrosine kinases in addition to the constitutively active Ser/Thr kinase MPSK1 (Eswaran et al., 2008) and in all Fes structures presented here (Figure S6). Second, binding of the substrate peptide also induces a β sheet secondary structure between the substrate peptide and a strand in the activation segment (βI), with typical main chain hydrogen bond interactions. The activation segment in active Fes showed significantly higher temperature factors than in either of the Fes-substrate complexes, indicating the stabilizing effect of substrate binding (Figure 3C). A similar antiparallel sheet interaction is also present in insulin receptor-substrate complexes, suggesting that stabilization of the activation segment by substrate binding is a more widespread mechanism of tyrosine kinase activation (Figure S6).

**Figure 4:**
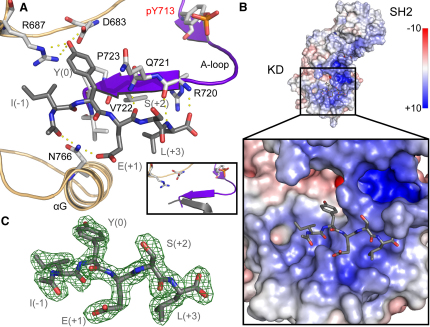
Substrate Interaction with the Fes Kinase Domain (A) Details of the substrate peptide (IYESL) interaction with the kinase domain. (B) Structure of the Fes-substrate complex showing a detail of the location of the peptide (shown in sticks). The surface has been colored by electrostatic potential between −10 and +10 kcal/mol. (C) 2F_o_ – F_c_ electron density map contoured at 2σ around the substrate peptide residues.

Fes is only distantly related to tyrosine kinases of known substrate specificity. We used a degenerate peptide library and a peptide array comprising all single substitutions of a known substrate peptide (EAEIYEAIE) to determine the peptide sequence preferentially recognized by the Fes active site (Figure S7). The two screens revealed that Fes prefers substrates with bulky aliphatic residues at the position N terminal to the substrate tyrosine (position −1), an acidic or phosphorylated residue at position +1, and hydrophobic residues at position +3. The structure of Fes in complex with its consensus peptide IYESL supports the peptide library results (Figures 4 and S7). Selectivity for hydrophobic interactions in substrate positions −1 and +3 can be explained by binding of the corresponding substrate residues isoleucine and leucine to hydrophobic pockets on the Fes kinase, and selectivity for acidic residues in position +1 is due to formation of a hydrogen bond with N766 located in helix αG. The determined substrate specificity corresponds well to known exogenous Fes-substrate sites (Greer, 2002).

### Fes Is Stabilized by Ligand Binding to Its SH2 Domain

**Figure 5:**
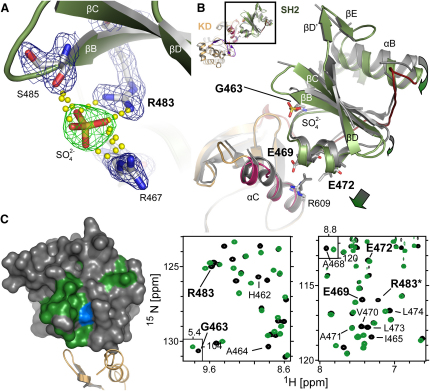
Stabilization of the Fes SH2 Domain by Ligand Binding (A) Coordination of a sulphate ion in the SH2 phosphotyrosine-binding site in active Fes and 2F_o_ – F_c_ electron density map contoured at 2σ around residues that coordinate the sulfate ion. (B) Superimposition of active Fes (green) and Fes phosphorylated at the activation segment Y713 with an unligated SH2 domain (gray). Structural changes associated with ligand binding to the SH2 domain are indicated by arrows. (C) Effect of the R/M mutation in the Fes SH2 domain measured by NMR. Overlay of ^1^H-^15^N HSQC spectra of wild-type (black) and mutant R/M (green) isolated Fes SH2 domain, with regions of the spectra relating to residues located in the SH2-kinase domain interface indicated in the right two panels (R483^∗^: side chain resonance). Residues that showed significant chemical shift changes in the spectra of the R/M mutant SH2 domain, as compared to wild-type, are depicted in green on the Fes SH2 domain surface in the left panel. Blue indicates the position of R483. Helix αC and the loop between strands β4 and β5 of the kinase domain are shown in orange.

In the latter two structures, crystallized in the absence of phosphomimetic salt ions, there was no ligand bound to the SH2 domain. A striking feature of both structures was the large degree of disorder in SH2 domain loop regions as well as in the loop connecting the sheet β3 with αC. In addition, helix αC showed very high temperature factors, indicating a high degree of mobility of this regulatory element (Figures 3C and 5B). In contrast, the sulphate ion occupying the pTyr-binding site of the SH2 domain in the active Fes structure formed a network of salt bridges typically observed in SH2 domain phosphopeptide complexes, correlating with stabilization and ordering of the SH2 domain and αC (Figures 5A and 5B). The high flexibility of residues in the SH2-kinase domain interface in the absence of an SH2 ligand suggested that Fes activity and SH2 domain ligand binding are coupled. To pursue this model, we substituted R483 in the SH2 pTyr-binding pocket with methionine (mutant R/M), which abolishes pTyr recognition (Figure S4A). The R/M mutant failed to autophosphorylate in transfected cells and had a significantly reduced ability to transphosphorylate cortactin (Figures 2A and 2B). We used NMR spectroscopy to assess whether this substitution might affect SH2 residues at the interface with the kinase domain. An HSQC NMR spectrum of an ^15^N-labeled sample of the isolated R/M mutant Fes SH2 domain revealed chemical shift changes of amide resonances of the critical interface residues G463 and E469 and nearby residues, as compared with the wild-type SH2 domain (Figure 5C). While these changes are ligand independent, the low activity of the R/M mutation, together with the structural data, suggests a coupling between SH2 domain pTyr binding and the catalytically active Fes conformation.

In the active Fes structure, the pTyr-binding site of the Fes SH2 domain is only about 30 Å distant from the active site of the kinase domain. Since peptides bound to the activation segment and the SH2 domain should be in the same N- to C-terminal orientation, the Fes phosphorylation site and the pTyr that recognize the Fes SH2 domain could, in principle, be connected within the same polypeptide chain by a short intervening sequence (see Figure 7). To test this model, we synthesized primed substrate peptides, each containing a C-terminal phosphorylated SH2 domain-binding site, linked by a polyglycine spacer of variable length to an unphosphorylated tyrosine located in a minimal Fes consensus substrate motif. We incubated these peptides with purified Fes SH2-kinase to determine whether there is an optimal spacing that favors phosphorylation of the latter site. A primed peptide with a linker of 10 glycine residues was a significantly better substrate than peptides with shorter or longer spacers (Figure S8). These data define a mechanism through which the Fes SH2 domain activates kinase function and recruits substrates. The SH2 domain, the kinase domain, and substrate apparently interact cooperatively to yield a functional Fes kinase complex.

### A Stimulatory SH2-Kinase Domain Interaction in the Abl Tyrosine Kinase

**Figure 6:**
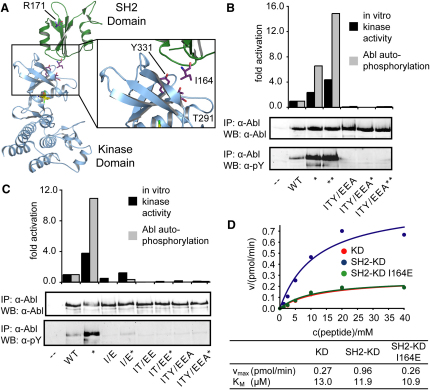
The Abl SH2 Domain Stimulates Kinase Activity (A) Ribbon representation of the active conformation of Abl (PDB entry 1OPL chain B) and close-up view of SH2-kinase domain interface. Critical interface residues (I164, T291, and Y331) are shown. R171 highlights the location of the pTyr-binding site in the SH2 domain. (B) SH2-kinase interface residues are required for in vivo Abl activity. HEK293 cells were transfected with c-Abl (WT) or the activated Abl variants PP (^∗^) or G2A/PP (^∗∗^) or with the corresponding ITY/EEA mutants, as indicated. Cells were lysed, and anti-Abl immunoprecipitates were analyzed by anti-Abl and anti-pTyr (pY) immunoblotting (lower panels). The histograph shows the in vitro kinase activity (mean of two experiments done in duplicate, black bars) and levels of autophosphorylation (mean of two immunoprecipitations, gray bars) of the Abl constructs relative to c-Abl and corrected for endogenous c-Abl levels. (C) I164 in the Abl SH2 domain is required for efficient kinase activity (WT = c-Abl, ^∗^ = Abl-PP; corresponding mutants are indicated). (D) The SH2 domain is necessary and sufficient to stimulate in vitro Abl kinase activity. Purified Abl proteins (see also Figures S9D and S9E) were evaluated for kinase activity in the presence of 50 μM ATP and the indicated concentrations of an optimal Abl substrate peptide. The K_m_ and v_max_ values are given below the graph.

Is the active SH2-kinase unit a broader feature of related cytoplasmic tyrosine kinases? To address this issue, we investigated the role of SH2-kinase interactions in determining the activity and substrate recognition of the pro-oncogenic c-Abl tyrosine kinase. In the autoinhibited conformation, the Abl SH2 domain docks onto the back of the C lobe of the kinase and, in conjunction with the SH3 domain, suppresses kinase activity (Hantschel et al., 2003; Nagar et al., 2003). Upon activation, the SH2 and kinase domains must be reoriented, and small angle X-ray scattering (SAXS) analysis of a constitutively active Abl variant (P242E/P249E, Abl-PP) has revealed that the SH3, SH2, and kinase domains are positioned in an extended arrangement (Nagar et al., 2006). Furthermore, crystallographic analysis identified a form of Abl consistent with the SAXS shape reconstruction, in which the SH2 domain forms an extensive interface with the N lobe of the kinase domain, with tight contacts formed between I164 in the SH2 domain and T291/Y331 in the kinase N lobe (Nagar et al., 2006; see Figure 6A). Simultaneous mutation of all three interface residues (I164E/T291E/Y331A [mutant ITY/EEA]) strongly reduced the activity of purified Abl proteins (Nagar et al., 2006) and abrogated Abl autophosphorylation and tyrosine phosphorylation of exogenous substrates in transfected cells, as well as ablating in vitro kinase activity, even in the presence of the most strongly activating Abl mutations (G2A/PP) (Figures 6B and S9A). However, since the two mutated N-lobe residues are close to the active site, these mutations could potentially cause an intrinsic defect in the kinase domain (Nagar et al., 2006). Indeed, substitution of either N-lobe residue alone (T291E or Y331A) impaired Abl kinase activity due either to disturbance of the SH2-kinase interface or a direct effect on catalytic activity (Figure S9C).

Therefore, we tested the effect of mutating the SH2 residue I164 to glutamate (mutant I/E) in the absence of further substitutions in the kinase N lobe by measuring Abl tyrosine kinase activity in transfected HEK293 cells and in vitro. For both wild-type c-Abl and activated Abl-PP, incorporation of the SH2 I164E mutation dramatically impaired Abl kinase activity and substrate phosphorylation, and only a slight further reduction was detected upon additional mutation of T291 and Y331 in the kinase domain (Figures 6C and S9B). These data suggest that full Abl activity requires the observed I164-mediated interaction of the SH2 domain with the kinase domain and that disruption of this interface severely compromises phosphorylation of Abl substrates in cells. Phosphotyrosine recognition by the SH2 domain may also modulate kinase activity since substitution of the conserved R171 in the SH2 domain with leucine, which abrogates pTyr binding, compromises Abl kinase activity and substrate phosphorylation (Figures S9B and S9C).

In contrast to the I164E mutation, substitution of Y158 in the SH2 domain with aspartate (mutant Y/D), which interferes with the autoinhibitory interaction of the SH2 domain with the kinase C lobe (Hantschel et al., 2003), increased the in vitro kinase activity of an Abl-PP mutant (Figure S9C). These data support the concept that the Abl SH2 domain interacts with the C lobe and N lobe of the kinase through distinct surfaces with opposing effects on catalytic activity.

To test whether the positive effect of the SH2 domain on catalytic activity is an intrinsic property of the SH2-kinase unit or requires additional Abl sequences, we compared the in vitro activity of the isolated c-Abl kinase domain (KD) to a polypeptide containing the SH2 and kinase domains (SH2-KD) (Figures S9D and S9E). HA-tagged Abl proteins were precipitated from HEK293 cells and assayed for phosphorylation of an optimal Abl substrate peptide. The SH2-KD protein was almost 4-fold more active than the kinase domain alone due to an effect on v_max_ (Figure 6D). This stimulatory effect of the SH2 domain was entirely suppressed by inclusion of the I164E SH2 mutation. These results argue that, as in Fes, a specific SH2-kinase interaction is required for efficient Abl catalytic activity and substrate phosphorylation in cells.

## Discussion

### An Activating Role of the Fes SH2 Domain

The SH2 domain was identified in the v-Fps oncoprotein as a noncatalytic module that exerts positive effects on the activity and substrate specificity of the adjacent tyrosine kinase domain (Sadowski et al., 1986; Stone et al., 1984). The structure of the active Fes SH2-kinase unit reveals tight packing of the SH2 domain with the kinase domain. This interaction is additionally maintained by electrostatic interactions between the SH2 helix αA and kinase helix αC, which position and stabilize αC in an active conformation. Mutations that disrupt this SH2-kinase interface, including targeted substitutions in Fes as well as the RX15m and AX9m insertion mutants in v-Fps, impair or eliminate kinase activity.

Stabilization of the kinase αC helix by interactions with helical elements is a common regulatory mechanism for protein kinases (Lei et al., 2005; Zhang et al., 2006). Here, we report a new mode of activation in which αC is positioned by a helix present in the SH2 domain; unusually, the activating helix is not aligned parallel with αC but is oriented perpendicular to it, maintained by long-range electrostatic interactions. The importance of the interaction between SH2 αA and kinase αC is directly shown by the functional rescue of a kinase-deficient mutant by reversing the charge polarity of residues at the interface.

In contrast to the active conformation, Fes with an unligated SH2 domain is partially disordered, containing unstructured regions in SH2 domain loops and a highly mobile helix αC. We infer that binding of a phosphorylated ligand to the SH2 domain stabilizes the SH2-kinase interaction and, thereby, the active conformation of the catalytic domain. Our data also suggest that the SH2 domain can play a role in substrate recognition by binding a phosphorylated target and orienting a substrate tyrosine to the active site. Fes substrates such as cortactin and HS1 have multiple pTyr sites separated by spacers of a suitable length for such docking-dependent phosphorylation. In support of this scheme, genetic and cellular data indicate that Fps/Fes proteins act downstream of, or in conjunction with, Src family kinases, which could generate primed sites for Fes activation (Murray et al., 2006; Udell et al., 2006).

### Coordinate Effects of SH2-Kinase Interactions, Autophosphorylation, and Substrate Recognition in Fes Activation

**Figure 7:**
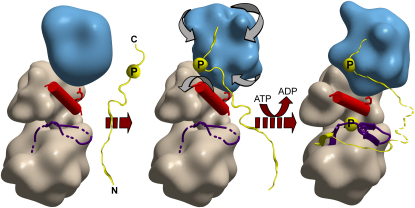
Cartoon Representation of Fes Activation In its unligated and unphosphorylated state, the Fes SH2 domain (blue), αC (red), and activation segment (purple) are significantly disordered (left). Binding of a primed peptide (yellow) stabilizes the SH2 domain, leading to a productive orientation of the SH2 domain, with respect to the kinase domain, and stable positioning of αC (middle). Phosphorylation of the activation segment at Y713 and binding of the substrate molecule to the kinase domain stabilizes the activation segment in a conformation suitable for catalysis (right).

A schematic model of Fes activation is shown in Figure 7. In the inactive state, we propose that the SH2 domain and the N lobe of the kinase domain are significantly disordered, and, as a consequence, the αC helix of the kinase N lobe is not optimally positioned for catalysis. In addition, the activation segment of the kinase domain is relatively unstable. However, localization to specific membrane sites promotes the active form of the kinase through a series of linked devices. For one, clustering induces intermolecular autophosphorylation, which stabilizes an active conformation of the activation loop. In addition, juxtaposition with potential targets allows a primed substrate or scaffold to bind and stabilize the SH2 domain, thereby promoting a productive interaction with the kinase N lobe that locks the αC helix in an active configuration. The active state could be further stabilized by association of a substrate peptide with the activation loop of the kinase domain. According to this model, Fes acts as a coincidence detector that is only fully activated by the combined effects of its interactions with membrane, a pTyr docking site, and substrate. Fes may, thereby, achieve substrate selectivity in part by coupling target recognition to kinase activation.

The N-terminal Fps/Fes F-BAR domain forms oligomers and interacts with phospholipids (Greer, 2002; Tsujita et al., 2006), and the F-BAR domains of PCH proteins make extended filaments implicated in membrane tubulation and endocytosis (Frost et al., 2008). We speculate that recruitment of the F-BAR domain to the membrane clusters Fps/Fes proteins in a fashion that promotes autophosphorylation, and it positions the SH2-kinase unit to phosphorylate targets such as cortactin that are involved in cytoskeletal reorganization and vesicular trafficking.

### Regulatory Roles of the SH2 Domain

The SH2 domains of cytoplasmic tyrosine kinases can have two general types of regulatory effects. In the kinase-active state, the SH2 domain can direct subcellular localization and substrate recruitment and promote an active conformation of the adjacent catalytic domain. The Fes kinase appears optimized for this positive mode of regulation. Conversely, the SH2 domain of kinases such as Src, Abl, and ZAP-70 can act in conjunction with an additional SH2 or SH3 domain to maintain an inactive state through intramolecular interactions with the catalytic domain (Figure S10). However, in these latter kinases, the SH2 domain is bifunctional in the sense that it is also critical for active signaling. For example, upon activation, the Abl SH2 and SH3 domains redistribute from their autoinhibitory positions on the backside of the kinase domain to adopt an extended conformation, with the SH2 domain contacting the tip of the kinase N-terminal lobe through a conserved isoleucine residue. We find that this latter interface allows the Abl SH2 domain to stimulate catalytic activity. The Abl SH2 domain binds primed substrates such as p130^cas^ and enhances their processive phosphorylation (Mayer et al., 1995), suggesting that, as in Fes, the Abl SH2 domain has functions in promoting both substrate recognition and catalytic activity. These data are consistent with a more general role for the SH2-kinase unit in downstream signaling by cytoplasmic tyrosine kinases.

As with autoinhibitory interactions, the mechanisms by which the SH2 domain contributes to the active state likely vary from kinase to kinase. The SH2 domains of active Fes and Abl have distinct interfaces and orientations with respect to the kinase domain. In the active conformation of C-terminal Src kinase (Csk), the SH2 and SH3 domains bind on opposing sides of the kinase N lobe, and their deletion results in ∼100-fold reduction in the k_cat_ of Csk, demonstrating that the SH3-SH2 domains have an activating function (Ogawa et al., 2002; Sondhi and Cole, 1999). Interestingly, this effect is potentiated by phosphopeptide binding to the SH2 domain (Lin et al., 2006). In the case of the Tec family member Btk, a low-resolution structural analysis by SAXS has suggested an extended linear arrangement of domains (Marquez et al., 2003), while mutagenesis data suggest that the SH2 domain and SH2-kinase linker of Tec family members stabilize αC in an active conformation (Joseph et al., 2007). Finally, active structures of Src family kinases show little interaction with the catalytic domain; since the isolated kinase domain is fully active when phosphorylated, the SH2 domain may primarily target the kinase to an appropriate site and promote processive phosphorylation without directly influencing kinase activity (Cowan-Jacob et al., 2005; Scott and Miller, 2000). Thus, the relative orientation of the SH2 domain with respect to the kinase domain could define its preference for specific substrates or scaffolds and the extent to which SH2 domain ligands control kinase activity. Our data for Fes provide a precise molecular mechanism by which an SH2 domain stimulates kinase activity and substrate recognition, and we show that a functionally similar SH2-kinase unit is employed for active Abl signaling.

## Experimental Procedures

### Protein Expression and Purification

cDNAs encoding human Fes (NP_001996) were cloned into pNIC28-Bsa4. Expression constructs were transformed into phage-resistant *E. coli* BL21(DE3)-R3 cotransformed with an expression vector encoding *Yersinia* phosphatase YopH (plasmid kindly provided by J. Kuriyan) (Seeliger et al., 2005). Cells were grown and Fes SH2-kinase proteins were purified according to previously described procedures (see Supplemental Data for details).

### Protein Phosphorylation

Protein samples were dialyzed overnight (50 mM HEPES [pH 7.5], 500 mM NaCl, and 5% glycerol), and phosphorylation was carried out by addition of 10 mM DTT, 10 mM Na_3_VO_4_, 2 mM (NH_4_)_2_SO_4_, 10 mM MgCl_2_, 2 mM ATP, and 5 mM MnCl_2_. Phosphorylated Fes was purified by gel filtration, concentrated to 10 mg/ml, and used for crystallization. The identities of recombinant proteins were confirmed using electrospray mass spectrometry (ESI-TOF) (Agilent).

### Mammalian Expression Constructs

Wild-type p130^Gag-Fps^ and the RX15m and AX9m mutants (Stone et al., 1984) were subcloned into the vector pRK5-myc (Winberg et al., 2000). Full-length human Fes was cloned into the vector pCMV7.1-3xFlag (Sigma, St. Louis, MO). Site-directed mutagenesis was performed using the QuickChange® II XL Kit (Stratagene, La Jolla, CA) following the manufacturer's directions. A GST-tagged C-terminal fragment of human cortactin (residues 382–550, accession Q14247) was prepared by PCR from cDNA (accession BC008799) and cloned into pEBG vector (gift from C. Duckett, University of Michigan Medical School, Ann Arbor).

### Tissue Culture

HEK293T cells were maintained in Dulbecco's modified Eagle's medium supplemented with 10% (vol/vol) fetal bovine serum, 100 units/ml penicillin G sodium, and 100 μg/ml streptomycin and incubated at 37°C, 5% CO_2_.

### In Vivo Kinase Assay

Flag-tagged Fes or Myc-tagged p130^Gag-Fps^ and mutants thereof were cotransfected with GST-cortactin into HEK293T cells. After 24 hr, cells were lysed with NP-40 cell lysis buffer (50 mM Tris-HCl [pH 7.5], 150 mM NaCl, 1% NP-40, and 1 mM EDTA) that contained 1 mM phenylmethylsulfonyl fluoride, 10 μg/ml leupeptin, 10 μg/ml aprotinin, and 10 μg/ml pepstatin A. Cell lysates, normalized for protein concentration, were used in anti-Flag M2 antibody-agarose (Sigma) or anti-Myc antibody immunoprecipitations or glutathione sepharose affinity precipitations. Associated proteins were resolved using SDS-PAGE and analyzed by western blotting using anti-Flag M2 (Sigma), anti-c-Myc 9E10 (Santa Cruz), HRP-conjugated anti-GST (Santa Cruz), or anti-pTyr 4G10 (Upstate) antibodies.

### Peptide Synthesis

Synthetic peptides were prepared using Fmoc (9-fluorenyl methoxycarbonyl) solid-phase chemistry. Phosphotyrosine was incorporated using the N-fluorenylmethyloxycarbonyl-O-phospho-L-tyrosine derivative. Peptides were purified using reverse-phase HPLC, and the authenticity was confirmed by mass spectrometry.

### Crystallization and X-Ray Data Collection

Sitting drops were set up using a Mosquito crystallization robot (TTP Labtech, Royston UK). Staurosporine and peptides were added to a final concentration of 1 mM and 250–300 μM, respectively. The active form (I) was crystallized from 0.2 M Na_2_SO_4_, 0.1 M bis-tris propane (pH 7.5), 20% PEG 3350, and 10% ethylene glycol. The unphosphorylated form (II) gave crystals from 20% PEG 3350 and 0.1 M sodium malate in the presence of the peptide Ac-IYESL. The phosphorylated protein (III) gave crystals from 30% mPEG 2000 and 0.15 M NaBr in the presence of the peptide Ac-IYESL. Crystals were soaked in a cryoprotectant solution containing the crystallization condition supplemented by 25% ethylene glycol and were flash-frozen in liquid nitrogen. Data sets were collected at the X10 beam line (Swiss Light Source) using a MAR225 detector at a single wavelength of 0.9807 nm in the case of I or in house on a Rigaku FRE rotating anode equipped with a Rigaku HTC image plate detector in the cases of II and III.

### Data Processing, Molecular Replacement, and Refinement

Data were indexed and integrated using MOSFLM (Leslie and Powell, 2007) and were scaled using SCALA (Evans, 2007). The structure of I was determined using molecular replacement and PHASER (McCoy et al., 2005) using an ensemble comprising PDB accession codes 1OPL, 1OPK, and 2FO0. All structures were refined using REFMAC5 (Murshudov et al., 1997). Thermal motions were analyzed using TLSMD (Painter and Merritt, 2006), and hydrogen atoms were included in late refinement cycles. The models and structure factors have been deposited with PDB accession codes: 3BKB (active Fes [I]), 3CBL (Fes/Ac-IYESL [II]), and 3CD3 (pY713-Fes/Ac-IYESL [III]). Protein ribbon graphic representations were created using PYMOL (version 0.98) (DeLano, 2002).

## Accession Numbers

The models and structure factors of Fes have been deposited in the Protein Data Bank with the PDB accession codes 3BKB (active Fes), 3CBL (Fes/Ac-IYESL), and 3CD3 (pY713-Fes/Ac-IYESL).

## Supplemental Data

Document S1. Supplemental Experimental Procedures, One Table, and Ten Figures
