# Supplementary material for: Structural Coupling of SH2-Kinase Domains Links Fes and Abl Substrate Recognition and Kinase Activation
Source: Cell. 2008 Sep 5;134(5):793–803. doi: 10.1016/j.cell.2008.07.047 (PMC2572732; doi:10.1016/j.cell.2008.07.047)
Supplement: Document S1. Supplemental Experimental Procedures, One Table, and Ten Figures [file mmc1.pdf]

Cell, *Volume 134*

Supplemental Data

Structural Coupling of SH2-Kinase Domains Links Fes and Abl Substrate Recognition and Kinase Activation

Panagis Filippakopoulos, Michael Kofler, Oliver Hantschel, Gerald D. Gish, Florian Grebien, Eidarus Salah, Philipp Neudecker, Lewis E. Kay, Benjamin E. Turk, Giulio Superti-Furga, Tony Pawson, and Stefan Knapp

Supplemental Experimental Procedures

Protein expression and purification – Full-length cDNA encoding human Fes (NP_001996) was obtained from the mammalian gene collection (MGC) and cloned into pNIC28-Bsa4 using ligation independent cloning. Expression constructs were transformed into phage resistant *E. coli* BL21(DE3)-R3 co-transformed with an expression vector encoding *Yersinia* phosphatase YopH (plasmid kindly provided by J. Kuriyan) (Seeliger et al., 2005). Cells were grown in Terrific Broth medium (TB) containing 50 µg/ml kanamycin and 50 µg/ml streptomycin at 37 ^{o}C to an optical density of about 3.5 (OD_{600}) before the temperature was decreased to 18 ^{o}C for a 12h induction of protein expression (0.1 mM isopropyl-β-D-thiogalactopyranoside - IPTG).

Cells were re-suspended in lysis buffer (100 mM NaH_2PO_4, pH 8.0 at 25 ^{o}C, 1 M NaCl, 5% glycerol, 0.5 mM tris(2-carboxyethyl) phosphine (TCEP), 10 mM imidazole) in the presence of a protease inhibitor cocktail tablet mix (Complete, EDTA-free Protease Inhibitor Cocktail, Roche Diagnostics Ltd.) and lysed using an EmulsiFlex-C5 high pressure homogenizer (Avestin) at 4 ^{o}C. The lysate was incubated with 0.15% polyethylenimine (PEI) at pH 7.5 for 30 min at 4 ^{o}C in order to remove residual DNA, and cleared by centrifugation and filtration through a 0.2 µm serum Acrodisc filter.

Protein purification was carried out using an automated ÄKTAexpress system (GE/Amersham Biosciences) which contained a His-trap and a gel filtration column (Superdex 75 16/60 HiLoad, GE/Amersham Biosciences) at 7 ºC. The His-trap column was

washed with 50 ml binding buffer (50 mM NaH_2PO_4, 500 mM NaCl, 10 mM imidazole pH 8.0, 5% glycerol, 0.5 mM TCEP), 100 ml wash buffer (50 mM NaH_2PO_4, 500 mM NaCl, 20 mM imidazole, pH 8.0, 5% glycerol. 0.5 mM TCEP), and recombinant Fes was eluted using elution buffer (50 mM NaH_2PO_4, 500 mM NaCl, 300 mM imidazole pH 8.0, 5% glycerol, 0.5 mM TCEP). The peak fraction was further purified by size exclusion chromatography (SEC) using a Superdex 75 16/60 HiLoad gel filtration column (GE/Amersham Biosciences) equilibrated with SEC buffer (10 mM HEPES, pH 7.4, 500 mM NaCl, 5% glycerol, 10 mM dithiothreitol (DTT)). The protein was treated for 12 h at 4 ^{o}C with TEV protease to remove the hexa-histidine tag. The cleaved tag and uncleaved protein were removed using a nickel-nitrilotriacetic acid agarose column. Purity was monitored by SDS-polyacrylamide gel electrophoresis and final samples were concentrated to 15 mg/ml in SEC buffer.

Peptide Synthesis – Synthetic peptides were prepared using Fmoc (9-fluorenyl methoxycarbonyl) solid phase chemistry using either an ABI 433 (Applied Biosystems, Inc., Foster City, CA) or Prelude (Protein Technologies, Inc., Tucson AZ) peptide synthesizer. Phosphotyrosine was directly incorporated in the peptides using the N-fluorenylmethyloxycarbonyl-O-phospho-L-tyrosine derivative. Peptide cleavage from the resin and side-chain deprotection were achieved through a 90 min incubation at room temperature in trifluoroacetic acid, water and triisopropyl silane (95:2.5:2.5% by volume). Product was precipitated with cold t-butylethyl ether, collected by centrifugation and purified using reverse phase HPLC. The authenticity of each peptide was confirmed by mass spectrometry.

NMR Spectroscopy – Wild type or mutant R/M Fes SH2 domain (residues 448-553) were cloned into pGEX4T2 and expressed in *E. coli* BL21(DE3) grown in two times yeast tryptone (2xYT) medium containing 100 µg/ml ampicillin. Protein expression was induced at an optical density of about 1.3 (OD_{600}) with 1 mM IPTG for 4h at 37 ^{o}C. For ^{15}N-labeled NMR samples, M9 minimal medium, comprising 1 g/l $^{15}NH_4Cl$ was used. Lysis and protein purification was performed following the GE Healthcare manual for GST protein production. Purified GST fusion protein was finally dialyzed against 40 mM HEPES buffer, pH 7.5, 150

mM NaCl, 1 mM EDTA, 1 mM DTT. For NMR samples, the GST tag was cleaved by thrombin directly on the glutathione beads, according to standard protocols. ^{1}H-^{15}N-HSQC spectra of [U-^{15}N] wild type or mutant SH2 domains (1.2 mM in 20 mM Tris-HCl, pH 7.0, 100 mM NaCl, 1 mM DTT-d_{10}, 0.02% NaN_3, 10% D_2O) were recorded at 298 K on a Varian Unity INOVA 500 MHz NMR spectrometer equipped with a room temperature probe with z-axis pulsed field gradient capabilities. Sequence-specific ^{1}H and ^{15}N resonance assignments of the wild type Fes SH2 domain were obtained based on the resonance assignments of a shorter construct published previously (Scott et al., 2004) by the standard strategy (Wüthrich, 1986) using conventional ^{1}H-^{15}N heteronuclear NMR spectroscopy. To this end, a [^{1}H,^{15}N]-NOESY-HSQC spectrum (Zhang et al., 1994) with 120 ms mixing time was recorded on the same sample and spectrometer as above. All ^{1}H and ^{15}N backbone amide resonances from G447 (derived from cloning) to K553 with the single exception of D498 could be assigned unambiguously, plus the side-chain NH_2 groups of Q452, Q458, Q486, Q489, Q507, N511, Q534, and Q535, the $^{1}H\varepsilon$-$^{15}N\varepsilon$ guanidinium resonances of R483, R514, and R547, and the $^{1}H\varepsilon1$-$^{15}N\varepsilon1$ indole resonances of W460 and W497. In all NMR experiments the H_2O resonance was suppressed by gradient coherence selection with quadrature detection in the indirect ^{1}H and ^{15}N dimensions achieved by States-TPPI (Marion et al., 1989) and the echo-antiecho method (Kay et al., 1992; Schleucher et al., 1993), respectively. The spectra were processed with NMRPipe (Delaglio et al., 1995) software and analyzed with NMRViewJ 8.0.a15 (Johnson and Blevins, 1994) and SPARKY (Goddard and Kneller, 1996-2002). ^{1}H chemical shifts were referenced with respect to external DSS in D_2O and ^{15}N chemical shifts were referenced indirectly (Markley et al., 1998).

References:

Delaglio, F., Grzesiek, S., Vuister, G.W., Zhu, G., Pfeifer, J., and Bax, A. (1995). NMRPIPE - A multidimensional spectral processing system based on UNIX pipes. Journal of Biomolecular Nmr *6*, 277-293.

Goddard, T.D., and Kneller, D.G. (1996-2002). SPARKY 3 (University of California, San Francisco).

Johnson, B.A., and Blevins, R.A. (1994). NMR VIEW - A Computer-program for the visualization and analysis of NMR data. Journal of Biomolecular NMR *4*, 603-614.

Kay, L.E., Keifer, P., and Saarinen, T. (1992). Pure Absorption Gradient Enhanced Heteronuclear Single Quantum Correlation Spectroscopy with improved sensitivity. Journal of the American Chemical Society *114*, 10663-10665.

Marion, D., Ikura, M., Tschudin, R., and Bax, A. (1989). Rapid recording of 2D NMR-spectra without phase cycling - application to the study of hydrogen-exchange in proteins. Journal of Magnetic Resonance *85*, 393-399.

Markley, J.L., Bax, A., Arata, Y., Hilbers, C.W., Kaptein, R., Sykes, B.D., Wright, P.E., and Wuthrich, K. (1998). Recommendations for the presentation of NMR structures of proteins and nucleic acids - (IUPAC Recommendations 1998). Pure and Applied Chemistry *70*, 117-142.

Schleucher, J., Sattler, M., and Griesinger, C. (1993). Coherence selection by gradients without signal attenuation - application to the 3-dimensional HNCO experiment. Angewandte Chemie-International Edition in English *32*, 1489-1491.

Scott, A., Pantoja-Uceda, D., Koshiba, S., Inoue, M., Kigawa, T., Terada, T., Shirouzu, M., Tanaka, A., Sugano, S., Yokoyama, S*., et al.* (2004). NMR assignment of the SH2 domain from the human feline sarcoma oncogene FES. J Biomol NMR *30*, 463-464.

Seeliger, M.A., Young, M., Henderson, M.N., Pellicena, P., King, D.S., Falick, A.M., and Kuriyan, J. (2005). High yield bacterial expression of active c-Abl and c-Src tyrosine kinases. Protein Sci *14*, 3135-3139.

Wüthrich, K. (1986). NMR of proteins and nucleic acids (New York ; Chichester, Wiley).

Zhang, O., Kay, L.E., Olivier, J.P., and Forman-Kay, J.D. (1994). Backbone ^{1}H and ^{15}N resonance assignments of the N-terminal SH3 domain of drk in folded and unfolded states using enhanced-sensitivity pulsed field gradient NMR techniques. J Biomol NMR *4*, 845-858.

Supplemental Table I

Data collection and refinement statistics

Data collection	**Active Fes (I)**	**Fes/Ac-IYESL (II)**	**pFes/Ac-IYESL (III)**
PDB ID	3BKB	3CBL	3CD3
Space group	$P6_3$	$P2_12_12_1$	$P2_12_12_1$
Cell dimensions: a, b, c (Å)	80.4, 80.4, 131.4	35.7, 77.2, 149.5	35.4, 76.9, 150.6
Resolution* (Å)	1.78 (1.88-1.78)	1.75(1.84-1.75)	1.98 (2.09-1.98)
Unique observations*	46019 (6704)	42378 (6133)	29000 (4265)
Completeness* (%)	100.0 (100.0)	99.3 (100.0)	97.8 (100.0)
Redundancy*	14.1 (5.9)	3.3 (3.4)	3.5 (3.5)
Rmerge*	0.128 (0.787)	0.069 (0.541)	0.082 (0.472)
I/ σI*	18.7 (2.0)	12.3 (2.0)	10.2 (2.3)
Refinement	**I**	**II**	**III**
Resolution (Å)	1.78	1.75	1.98
R_{work} / R_{free} (%)	14.8/18.9	18.0/22.7	18.5/24.7
Number of atoms (protein/other/water)	2970/82/389	2871/105/328	2818/107/307
B-factors (Å^2) (protein/other/water)	27.7/27.7/36.4	39.6/30.1/45.7	48.9/42.5/53.7

* values in parentheses correspond to highest resolution shell

Supplemental Figure 1

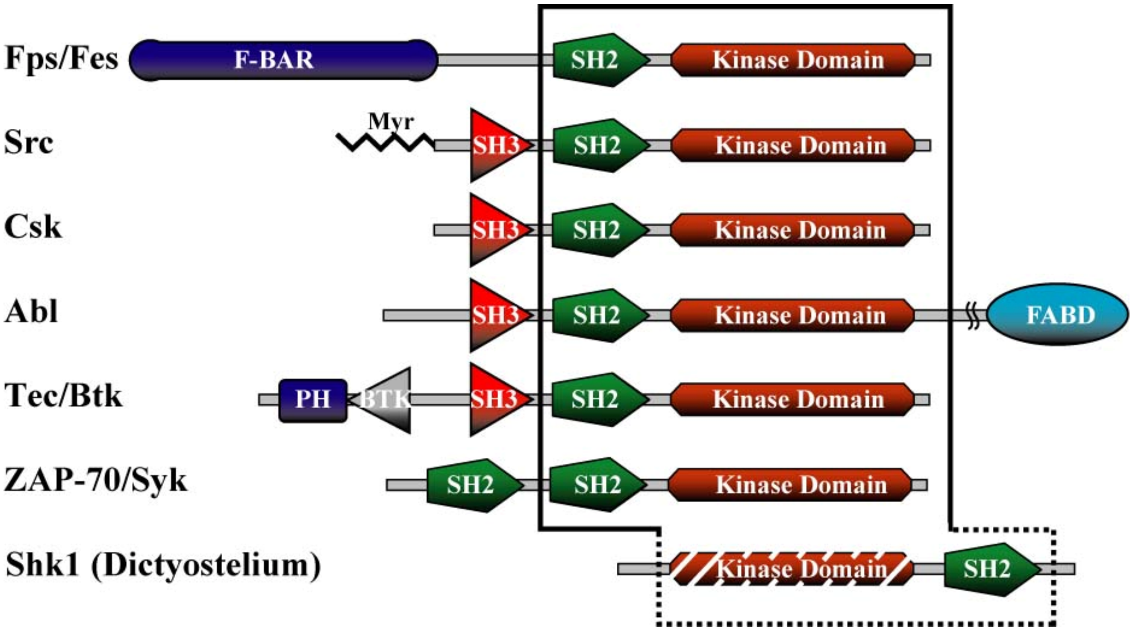

Supplemental Figure 1: The SH2-kinase domain combination is conserved in cytoplasmic tyrosine kinases. Examples of metazoan kinases with a core SH2-tyrosine kinase unit, flanked by various additional interaction domains, are shown. The domain architecture of the Shk1 protein kinase of *Dictyostelium discoideum* is also depicted. No bona fide tyrosine kinases have yet been identified in *Dictyostelium*, although tyrosine phosphorylation and phosphotyrosine-dependent SH2 domain recognition are present. The Shk1 kinase domain belongs to the TKL subfamily, and has dual specificity (Ser/Thr and Tyr) kinase activity *in vitro*.

Supplemental Figure 2

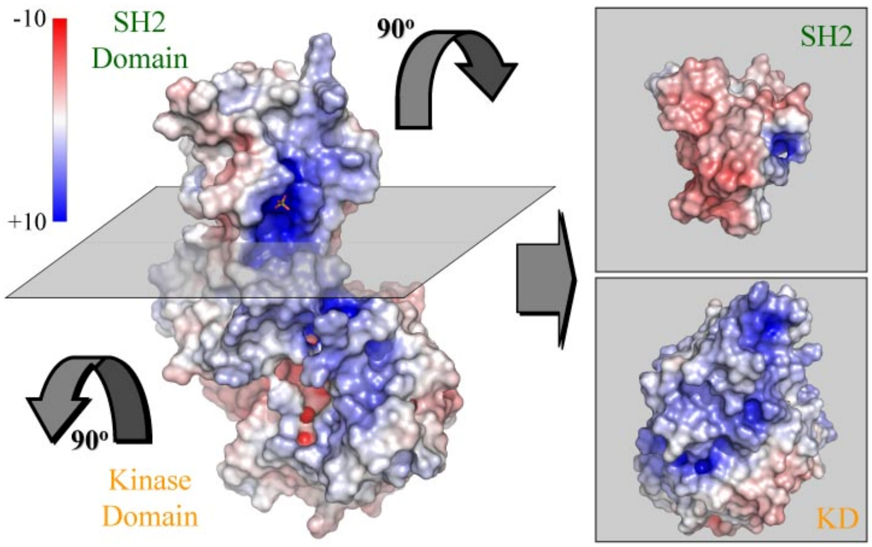

Supplemental Figure 2: Charge complementarity of the interface between the SH2 and kinase domains. Surfaces are colored by electrostatic potential between -10 and +10 kcal/mol. The SH2-kinase unit is shown to the left, with the site of the inter-domain interface sliced by a plane. To the right, each domain has been separated and rotated by 90° as indicated by the grey arrows, and the electrostatic potential of each interacting surface is shown. Note that the surface of the SH2 domain that interacts with the kinase domain (KD) is predominantly negatively charged, whereas the surface of the kinase N lobe that interacts with the SH2 domain is predominantly positively charged.

Supplemental Figure 3

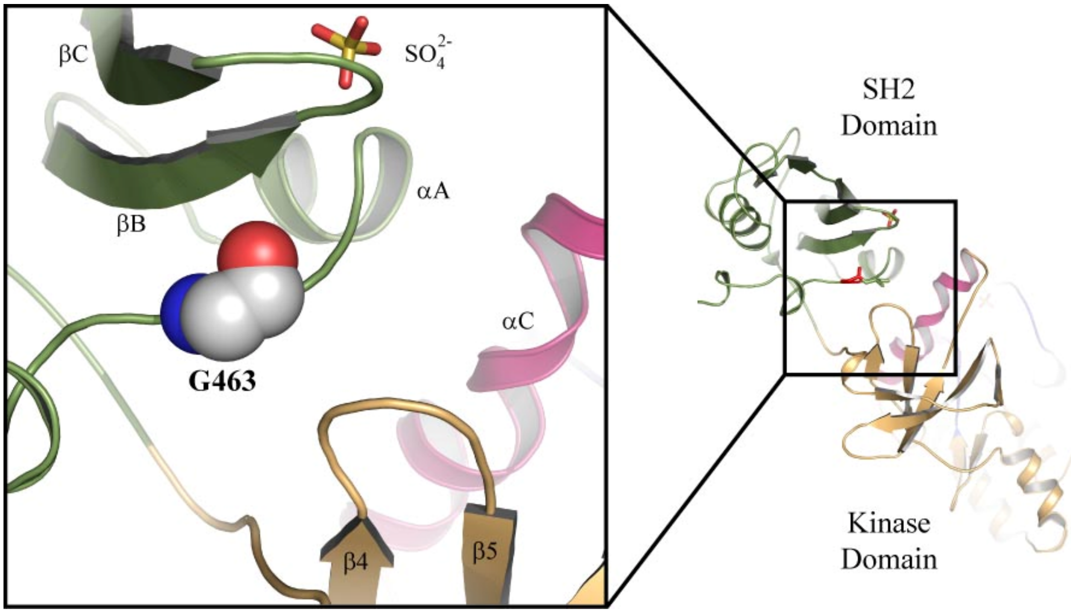

Supplemental Figure 3: Details of the SH2-kinase loop contact in the Fes SH2-kinase unit. The interface formed by the Fes SH2 loop region ^{462}HGAI^{465} and the loop between the kinase sheets β4 and β5 is shown. G463 is depicted as a CPK model.

Supplemental Figure 4

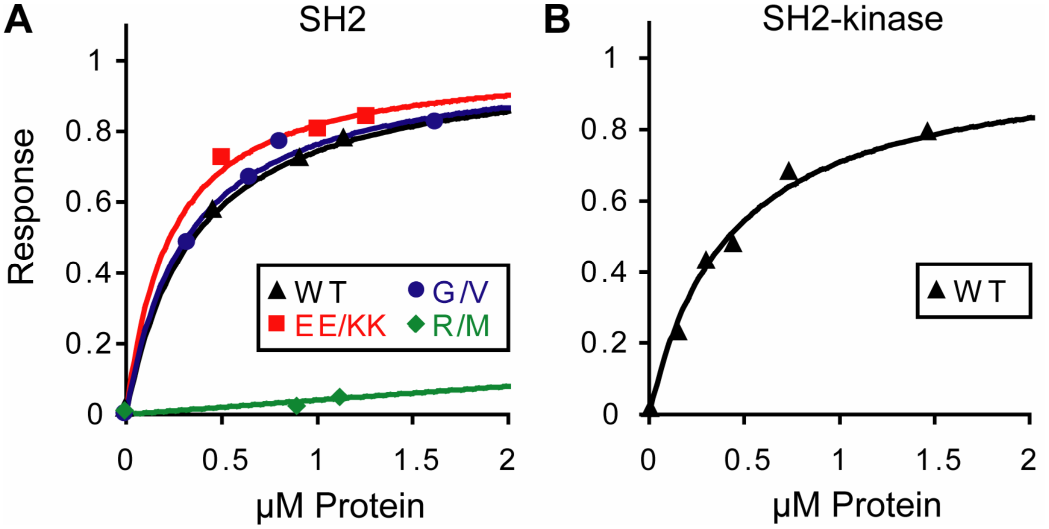

Supplemental Figure 4: Comparison of Fes SH2 and SH2-kinase binding to an ezrin-derived phosphopeptide (VpYEPVSY) as monitored by fluorescence polarization. **A:** Wild type Fes SH2 domain (triangles), the mutant G/V (spheres) and the mutant EE/KK (squares) have similar binding affinities (K_D ~400 nM) while the mutant R/M (diamonds) reveals only residual phosphopeptide binding. **B:** The Fes SH2-kinase has a similar phosphopeptide-binding affinity as the isolated SH2 domain (K_D ~400 nM). Fluorescence polarization binding studies were performed at 22 °C with 10 nM fluorescein-labeled ezrin peptide (fluorescein-VpYEPVSY-NH_2), in 40 mM HEPES buffer, pH 7.5, 150 mM NaCl, 1 mM EDTA, 1 mM DTT in the presence of increasing amounts of GST-SH2 domain or SH2-kinase domain. For the SH2-kinase the buffer was supplemented with 1 mM ATP and 5 mM $MgCl_2$. Data were fitted to a simple bimolecular binding model.

Supplemental Figure 5

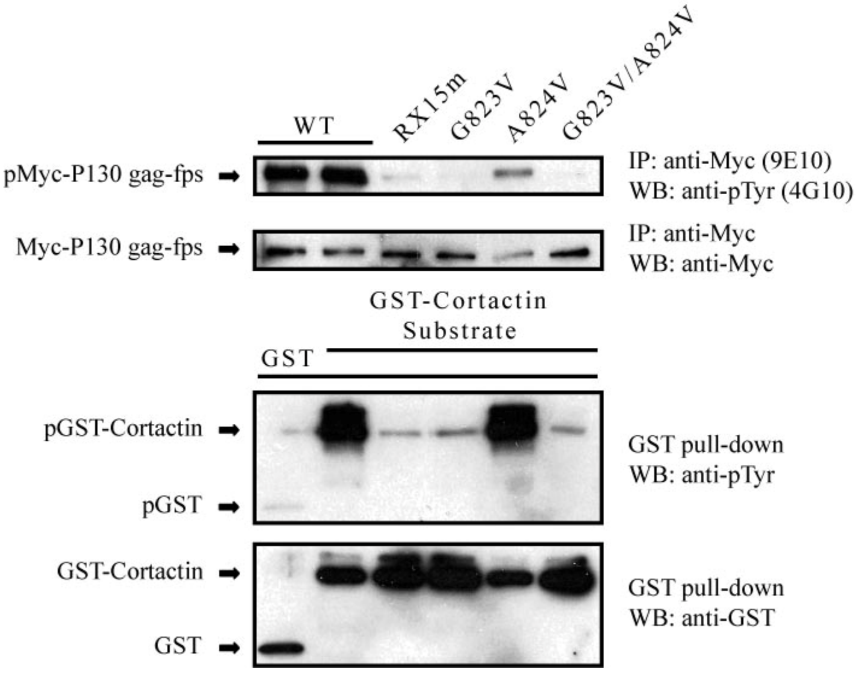

Supplemental Figure 5: Based on the crystal structure of human Fes, the indicated substitutions were introduced into the P130$^{gag\text{-}fps}$ oncoprotein, at residues predicted to lie at the interface of the N-terminal SH2 loop region and the kinase β4/β5 loop (G823 and A824, corresponding to G463 and A464 in human Fes). To assess the effects of these mutations on kinase activity, the indicated Myc-epitope-tagged P130$^{gag\text{-}fps}$ proteins were co-expressed with a GST-cortactin C-terminal substrate in human embryonic kidney cells (HEK293T). Cell lysates were prepared, protein concentrations determined, and equal amounts of protein were used in an anti-Myc antibody immunoprecipitation (IP) or glutathione-sepharose affinity precipitation. Following washing the associated proteins were resolved using 7.5% SDS-PAGE and analyzed by western blotting (WB) as indicated. In addition to novel mutations suggested by the structure of human Fes, we included the RX15m insertion mutant, which changes Y821 (corresponding to Y461 in human Fes) to SRD, for comparison.

Supplemental Figure 6

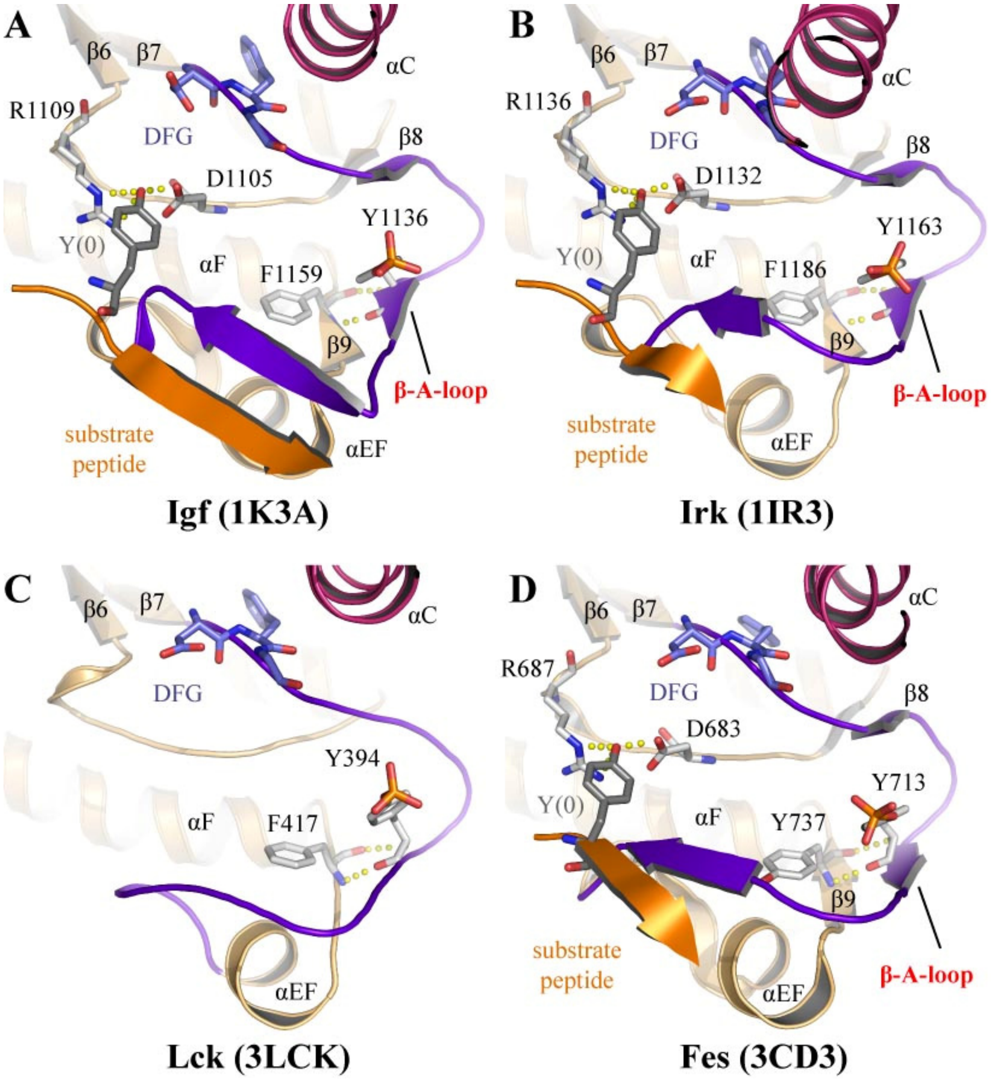

Supplemental Figure 6: Activation segment of active tyrosine kinases. Presence of activation segment anti-parallel sheets in active tyrosine kinases. Shown is the activation segment of insulin-like growth factor 1 receptor tyrosine kinase in complex with a substrate peptide (Favelyukis et al., 2001) (**A**), an insulin receptor-peptide complex (Hubbard, 1997) (**B**), active Lck (Yamaguchi and Hendrickson, 1996) in the absence of a substrate peptide (**C**) as well as the structure of the phosphorylated Fes-substrate complex (**D**). Hydrogen bonds in the activation segment sheets formed between the loop region connecting αEF and αF as well as the tip of the activation loop (β-A-loop) are shown as dotted lines, and the

peptide is shown as an orange ribbon. In Lck the hydrogen bonds are conserved but an anti-parallel sheet is not formed probably due to the absence of a stabilizing substrate molecule.

References:

Favelyukis, S., Till, J.H., Hubbard, S.R., and Miller, W.T. (2001). Structure and autoregulation of the insulin-like growth factor 1 receptor kinase. Nat Struct Biol *8*, 1058-1063.

Hubbard, S.R. (1997). Crystal structure of the activated insulin receptor tyrosine kinase in complex with peptide substrate and ATP analog. EMBO J *16*, 5572-5581.

Yamaguchi, H., and Hendrickson, W.A. (1996). Structural basis for activation of human lymphocyte kinase Lck upon tyrosine phosphorylation. Nature *384*, 484-489.

Supplemental Figure 7

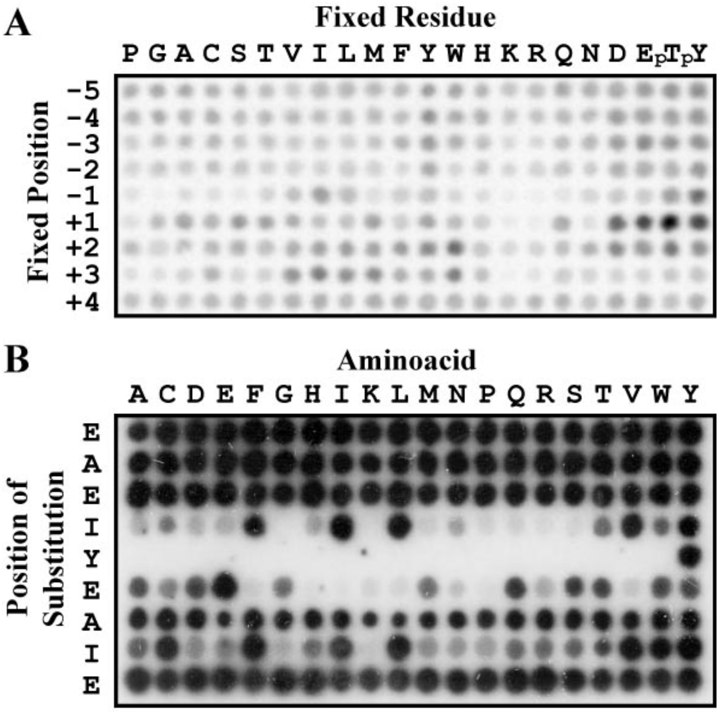

Supplemental Figure 7: Peptide array screening using either a degenerate peptide array library (**A**) or a spots blot kinase assay comprising all single substitutions of the substrate peptide EAEIYEAIE (**B**).

A: Phosphorylation motifs for Fes SH2-kinase were determined using a positional scanning peptide library approach essentially as described before but modified to allow for analysis of tyrosine kinases (Hutti et al., 2004). Reactions were carried out in multiwell plates in 50 mM HEPES, pH 7.4, 10 mM $MgCl_2$, 1 mM DTT, 0.1% Tween 20, 100 µM ATP (including 0.3 µCi/µl γ-[^{33}P]-ATP), 50 µM peptide substrate, and 0.1 – 0.2 µg/ml of phosphorylated Fes SH2-kinase for 2 hours at 30 ºC. Peptide substrates had the general sequence GAXXXXX-Y-XXXXAGKK-(biotin), where K-(biotin) is ε-(biotinamidocaproyl)lysine, and X is a roughly equimolar mixture of the 18 amino acids excluding cysteine and tyrosine residues. Each well contained a distinct peptide in which one of the X positions was replaced with one of 22 residues (one of the unmodified proteogenic amino acids, phosphorylated threonine or phosphorylated tyrosine). At the end of the incubation time, aliquots of each reaction were spotted onto streptavidin membrane, which was processed as described (Hutti et al., 2004).

B: A peptide array comprising all single substitutions of the substrate peptide EAEIYEAIE (Gish et al., 1995) was synthesized on a cellulose support using standard F-moc chemistry and an automated Multipep synthesizer (Intavis) as previously described (Wiesner et al., 2006). An additional glycine at the C terminus increased the linker length to the cellulose support. The membrane was first rinsed in 95% EtOH followed by several washing steps , 3x 5 min with TBS-T, 2x 5 min with TBS, and 2x 5 min with kinase reaction buffer (KRB – 250 mM NaCl, 20 mM $MgCl_2$, 20 mM $MnCl_2$ in 50 mM HEPES buffer pH 7.5). For the kinase reaction, 50 µg of purified Fes SH2-kinase was used in 5 mL of KRB containing 50 µCi of radio-labeled γ^{32}P-ATP and 40 µM unlabelled ATP. The reaction was stopped by three 20 min washes in Wash Buffer A (8M urea, 1% SDS, 0.5% beta-mercaptoethanol) followed by one 20 min wash in Wash Buffer B (50% EtOH, 10% acetic acid). The membrane was rinsed in 95% EtOH and allowed to air dry prior to exposing XAR film (Kodak) (Warner et al., 2008).

References:

Hutti, J.E., Jarrell, E.T., Chang, J.D., Abbott, D.W., Storz, P., Toker, A., Cantley, L.C., and Turk, B.E. (2004). A rapid method for determining protein kinase phosphorylation specificity. Nat Methods *1*, 27-29.

Wiesner, S., Wybenga-Groot, L.E., Warner, N., Lin, H., Pawson, T., Forman-Kay, J.D., and Sicheri, F. (2006). A change in conformational dynamics underlies the activation of Eph receptor tyrosine kinases. EMBO J *25*, 4686-4696

Gish, G., McGlone, M.L., Pawson, T., and Adams, J.A. (1995). Bacterial expression, purification and preliminary kinetic description of the kinase domain of v-fps. Protein engineering *8*, 609-614.

Warner N., Wybenga-Groot L.E., and Pawson T. (2008) Analysis of EphA4 receptor tyrosine kinase substrate specificity using peptide-based arrays. FEBS J *275*,2561-73.

Supplemental Figure 8

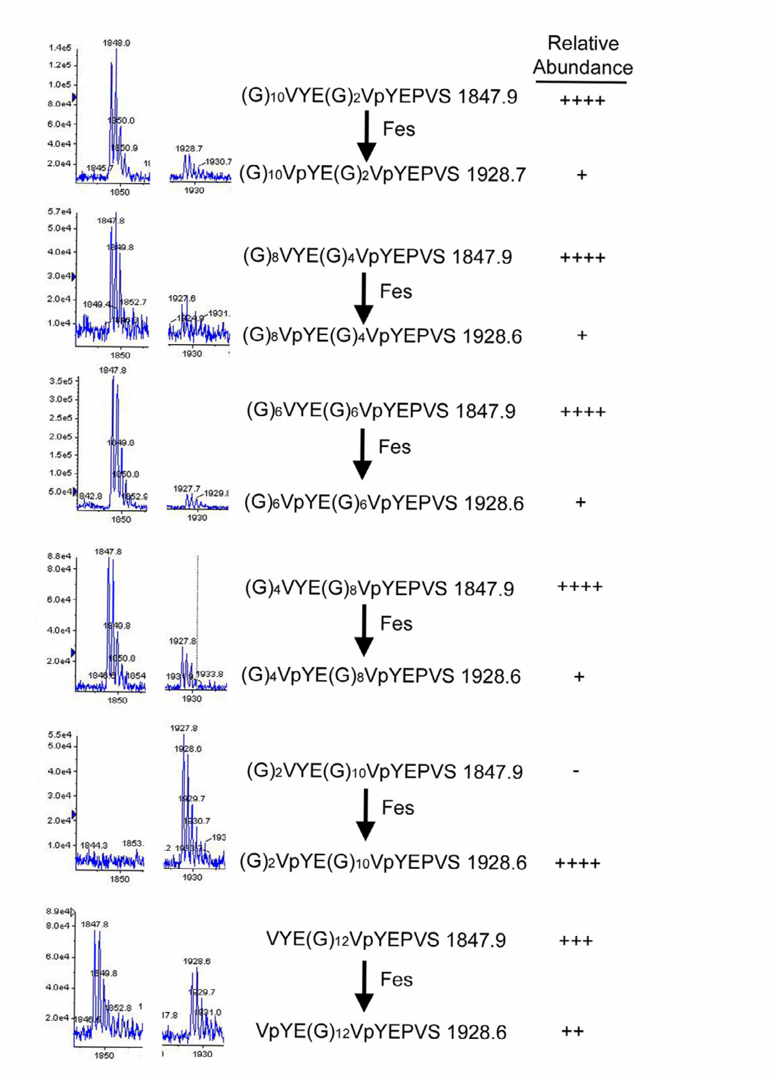

Supplemental Figure 8: Phosphorylation by purified Fes SH2-kinase of substrate peptides containing an SH2 domain-binding site (VpYEPVS) linked to an N-terminal partial Fes consensus site (VYE) via a flexible glycine linker. Phosphorylation of the N-terminal site was monitored by mass spectrometry after a 10 minute incubation of 100 μM peptide with Fes SH2-kinase and 4 mM ATP. The size and mass of the substrate peptide was kept constant by compensating for the increasing glycine chain length in the linker by shortening an N-terminal glycine tail. A spacer of 10 glycine residues yielded a peptide that was optimal as a substrate, as indicated by Fes dependent phosphate incorporation. Peaks of the peptides in

which the N-terminal tyrosine is unphosphorylated are to the left, and peaks of their phosphorylated counterparts are to the right.

Supplemental Figure 9

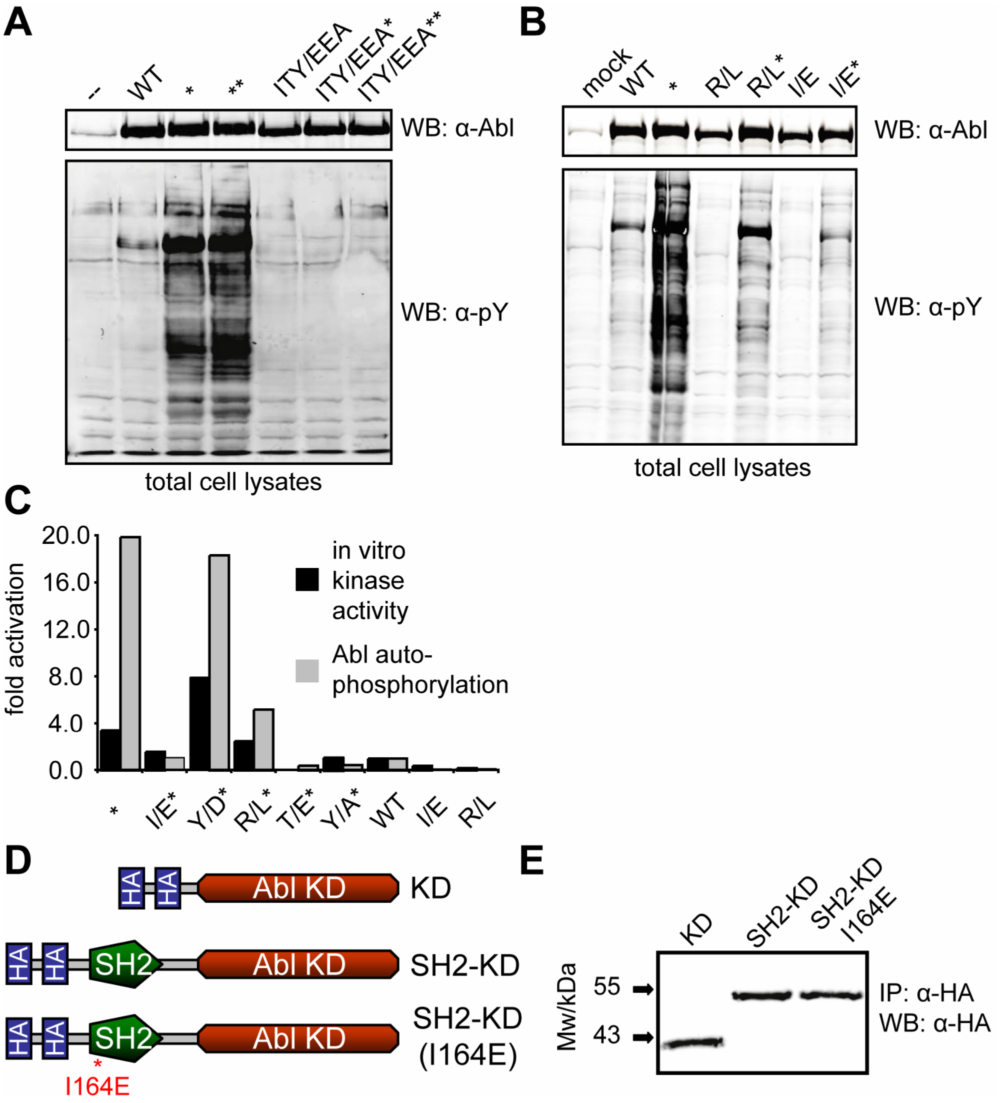

Supplemental Figure 9: Effects of SH2 and kinase domain mutations on Abl kinase activity.

A: I164E/T291E/Y331A triple substitution abrogates Abl-induced tyrosine phosphorylation in cells. HEK293 cells were transfected with c-Abl (WT), the activated Abl variants PP (*), G2A/PP (**), or the I164E/T291E/Y331A (ITY/EEA) triple derivatives of the WT and activated Abl proteins. Total cell lysates were immunoblotted with antibodies to Abl or phosphotyrosine (pY). Untransfected cell lysate is shown as a control (--).

B: Effects of SH2 domain mutations on protein tyrosine phosphorylation. Lysates of HEK293 cells transfected with c-Abl (WT), activated Abl variant PP (*), or WT and PP(*) derivatives

containing I164E (I/E) or R171L (R/L) mutations, were immunoblotted as described in **A**. A mock-transfected cell lysate is shown as a control (mock).

C: Effects of SH2 domain mutations on Abl kinase *in vitro* activity. The histogram shows the *in vitro* kinase activity (mean of two experiments done in duplicate, black bars) and levels of Abl autophosphorylation in cells (mean of two immunoprecipitations, grey bars) of the indicated Abl constructs relative to c-Abl and corrected for endogenous c-Abl levels. Y/D*, T/E* and Y/A* stand for the mutations Y158D, T291E, and Y331A, repectively, in the background of the activated Abl variant PP.

D,E Abl constructs and mutants (used in the kinase assay shown in Figure 6D) were immunoprecipitated using HA-agarose and immunoblotted with an anti-HA antibody (**E**).

DNA constructs – pSGT vector and pSGT-Abl constructs were previously described (Barila and Superti-Furga, 1998). To generate the Abl KD construct, a fragment containing amino acid residues 248-534 of human Abl 1b was amplified by PCR. For the SH2-KD construct, a fragment encompassing residues 138-534 was used. Both fragments were expressed as N-terminal HA-fusion proteins from pcDNA3.1. For clarity, numbering of amino acid residues in these constructs was retained according to full-length human Abl 1b. All point mutations were obtained using the quick-change site directed mutagenesis kit (Stratagene) and pSGT-Abl 1b, pcDNA3.1 HA-Abl-KD or pcDNA3.1 HA-Abl-SH2-KD as templates. All mutations were confirmed by sequencing.

Transfection and immunoprecipitation – Transfection of HEK293 cells and immunoprecipitation of Abl protein was carried out as described previously (Pluk et al., 2002). The relative concentration of immunoprecipitated Abl protein was determined by quantitative immunoblotting using the Li-cor Odyssey system and normalized for c-Abl wild type.

Kinase assays – Transfection of HEK293 cells, immunoprecipitation of Abl protein and Abl *in vitro* kinase assay was carried out as described previously (Hantschel et al., 2003; Pluk et al., 2002). The relative concentration of immunoprecipitated Abl protein and Abl

autophosphorylation was determined by immunoblotting (anti-Abl Ab-3 (Oncogene Science, Cambridge, MA), anti-HA (Rockland, Gilbertsville, PA) and anti-pTyr, 4G10 (Millipore, Billerica, MA)) and subsequent quantification using the Li-cor Odyssey system and normalized for c-Abl wild type.

References:

Barila, D., and Superti-Furga, G. (1998). An intramolecular SH3-domain interaction regulates c-Abl activity. Nat Genet *18*, 280-282.

Hantschel, O., Nagar, B., Guettler, S., Kretzschmar, J., Dorey, K., Kuriyan, J., and Superti-Furga, G. (2003). A myristoyl/phosphotyrosine switch regulates c-Abl. Cell *112*, 845-857.

Pluk, H., Dorey, K., and Superti-Furga, G. (2002). Autoinhibition of c-Abl. Cell *108*, 247-259.

Supplemental Figure 10

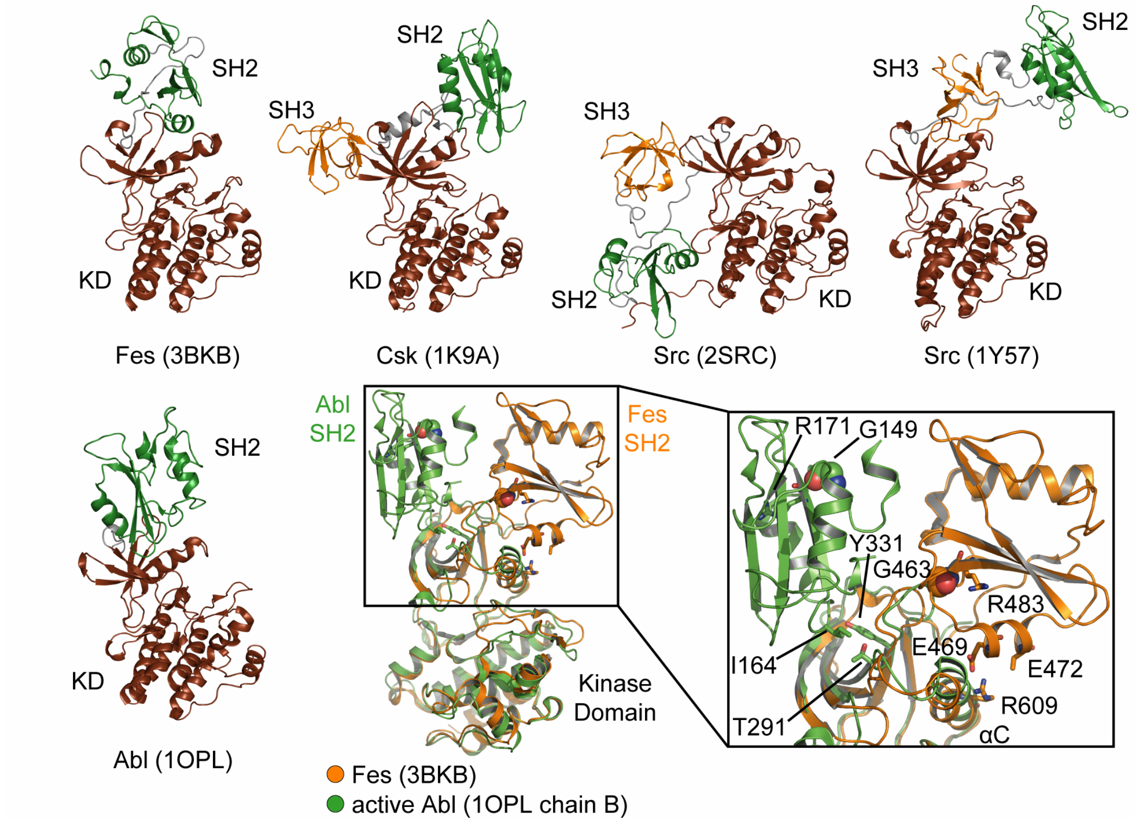

Supplemental Figure 10: Different types of domain orientation found in the SH2-kinase unit of cytoplasmic tyrosine kinases. A gallery of structures in ribbon representation is shown, including Fes, Csk (Ogawa et al., 2002), the inactive closed form of Src (Xu et al., 1999), the open form of Src (Cowan-Jacob et al., 2005) and the active form of Abl (Nagar et al., 2006). All structures have been aligned at the lower lobe of the kinase domain and are shown in identical orientation. Kinase domains (KD) are shown in red, SH2 domains in green and SH3 domains in orange. Also illustrated is a superimposition of Fes on the active Abl structure. The expanded frame shows the kinase-SH2 interacting regions, the key interface residues, and SH2 domain arginine residues involved in phosphotyrosine binding.

References

Cowan-Jacob, S.W., Fendrich, G., Manley, P.W., Jahnke, W., Fabbro, D., Liebetanz, J., and Meyer, T. (2005). The crystal structure of a c-Src complex in an active conformation suggests possible steps in c-Src activation. Structure *13*, 861-871.

Nagar, B., Hantschel, O., Seeliger, M., Davies, J.M., Weis, W.I., Superti-Furga, G., and Kuriyan, J. (2006). Organization of the SH3-SH2 unit in active and inactive forms on the c-Abl tyrosine kinase. Mol Cell *21*, 787-798.

Ogawa, A., Takayama, Y., Sakai, H., Chong, K.T., Takeuchi, S., Nakagawa, A., Nada, S., Okada, M., and Tsukihara, T. (2002). Structure of the carboxyl-terminal Src kinase, Csk. J Biol Chem *277*, 14351-14354.

Xu, W., Doshi, A., Lei, M., Eck, M.J., and Harrison, S.C. (1999). Crystal structures of c-Src reveal features of its autoinhibitory mechanism. Mol Cell *3*, 629-638.
